# Wild edible plants in Yeşilli (Mardin-Turkey), a multicultural area

**DOI:** 10.1186/s13002-019-0327-y

**Published:** 2019-11-05

**Authors:** Yeter Yeşil, Mahmut Çelik, Bahattin Yılmaz

**Affiliations:** Faculty of Pharmacy, Department of Pharmaceutical Botany, Istanbul University, Fatih, 34116 Istanbul, Turkey

**Keywords:** Ethnobotany, Wild edible plants, Cultural Importance index, Yeşilli, Mardin

## Abstract

**Background:**

The Yeşilli district (Mardin) is located in the southeastern of Turkey and hosts different cultures. The objective of this study was to record the traditional knowledge of wild edible plants used by indigenous people in Yeşilli, where no ethnobotanical studies have been conducted previously.

**Methods:**

An ethnobotanical study was carried out in Yeşilli district in March 2017–March 2019 to document the traditional knowledge of wild edible plants. The data were collected by interviewing 62 informants. Additionally, the data were analysed based on the cultural importance index (CI) and factor informant consensus (*F*_İC_) to determine the cultural significance of wild edible plants and knowledge of wild edible plants among the informants.

**Results:**

We documented 74 wild edible taxa belonging to 31 families and 57 genera in the present study. The richness of the wild edible taxa was highest for vegetables (46 taxa), followed by medicinal plants (17 taxa) and fruit (14 taxa). The most important families were Asteraceae (ten taxa), Rosaceae (seven taxa) and Fabaceae (six taxa). The most culturally important taxa (based on the CI index) were *Ficus carica* subsp. *carica*, *Lepidium draba*, *Anchusa strigosa*, *Rhus coriaria*, *Glycyrrhiza glabra*, *Sinapis alba*, *Gundelia tournefortii*, *Notobasis syriaca*, *Onopordum carduchorum*, *Malva neglecta*, *Mentha longifolia*, *Juglans regia* and *Urtica dioica*. The maximum number of use reports was recorded for vegetables (1011). The factor informant consensus index (*F*_*ic*_) varied between 0.95 and 0.98 for preserved vegetables, beverages and spices and processed fruits have the highest *F*_*ic*_ (0.99). We reported for the first time the ethnobotanical usage of 12 taxa as food. We also recorded the use of *Allium wendelboanum*, an endemic species in the study area.

**Conclusion:**

The obtained data were compared with data from other wild edible and ethnobotanical studies conducted in Turkey and particularly those conducted in eastern Turkey. Furthermore, the data were compared with data from studies conducted in the bordering countries of Iraq and Armenia. The present study reflects the cultural diversity of the region, and it is necessary to conduct more studies since it is thought that this diversity will contribute to the economy. This study will enable the traditional use of wild plants as food sources to be passed on to future generations.

## Introduction

Traditional knowledge of plants and their uses is the result of thousands of years of experience. The relevance of this knowledge to the increased daily living standards of rural populations as well as to decisions regarding the sustainable use of plant resources has frequently been noted [1].

Wild edible plants are defined as non-cultivated and non-domesticated plants [2]. Interest in the use of wild food sources has increased in recent years. Valuable nutritional supplements, including trace elements, vitamins and minerals, are acquired from many wild plants [3]. These supplements are known as ‘nutraceuticals’, and their importance has recently been highlighted [2].

In recent years, detailed information about wild food plants in Europe has been reviewed [2–4]. According to these studies, most of the plants that were previously used as food are used rarely or not at all today. However, while the traditional use of wild edible plants has greatly reduced due to socio-economic and ecological changes, wild plants are becoming a part of a new way of thinking about food: they are very important in healthy food, food safety and slow food movements. Ethnobotanical studies conducted in various parts of Europe have prevented the use of wild plants as food [5–12].

The Turkish flora comprises 8796 species, listed in ‘Flora of Turkey’ [13–15]. Additionally, according to ‘Illustrated Flora of Turkey’ [16–18], the most recent checklist [19] and publications about Turkish flora include 11,700 taxa. The endemism rate is 34% [17]. The Anatolian people, who have been living in this area for centuries have benefited from this rich diversity of plants primarily for food, medicine and other purposes [20]. However, systematic ethnobotanical studies only began in the mid-1990s [21] in Turkey. In rural areas, the people acquire the plants they need by gathering them from the surrounding mountains and forests [22–24].

Numerous studies on ethnobotanical and edible plants have been conducted in various regions of Turkey [25–94]. However, the southeastern region of Turkey, which was the focus of the present research, has been the subject of only a limited number of ethnobotanical studies [26–28, 31, 52, 60]. Several previous ethnobotanical studies have described the traditional knowledge of plants close to the present study area [31, 60]. Additionally, ethnobotanical studies have been conducted in nearby regions with the same cultural structure as the Yeşilli district [95–99].

Mardin province is a region within the boundaries of the region called ancient Mesopotamia that has hosted many civilisations. Since the beginning of history, many people and religious communities have lived together in this region. The different denominations of Muslims, Christians, Jews and Yezidis who believe in Melek Taus and the heliolatry Shams make up the majority of Mardin city. The Sun Temple in the ancient city of Dara, the Temple of Zarathustra where Deyrulzafaran Monastery was built and the Tur Abdin region called Abidler Dagi are important places that show that the region has been a religious centre for thousands of years [100]. The multicultural structure of Mardin has continued to the present day; Kurds, Assyrians, Arabs and Yezidis currently live together in this area. For this reason, at least three languages are spoken in daily life [100, 101].

The Yeşilli district of Mardin city, which is home to a portion of Mardin’s diverse culture, consists of nine rural and five urban settlements. The cultural diversity of the district is reflected in the local ethnobotanical knowledge. Although agriculture has declined in many regions in Turkey, it continues in Yeşilli and because of this, the population is dense in rural areas, and there have not been botanical studies in this area. The focus of the present study was to identify wild edible plants, rather than agricultural plants. Yeşilli was selected because of its mix of cultures and the fact that no ethnobotanical or floristic studies had preciously been conducted in this region. We focused on (1) identifying edible plant use, (2) determining new uses of wild edible plants and evaluating our findings in terms of cultural ethnobotany by comparing with previous ethnobotanical studies and (3) transferring this knowledge to future generations and offering alternative sustainable sources of food.

## Materials and methods

### Study area

**Fig. 1:**
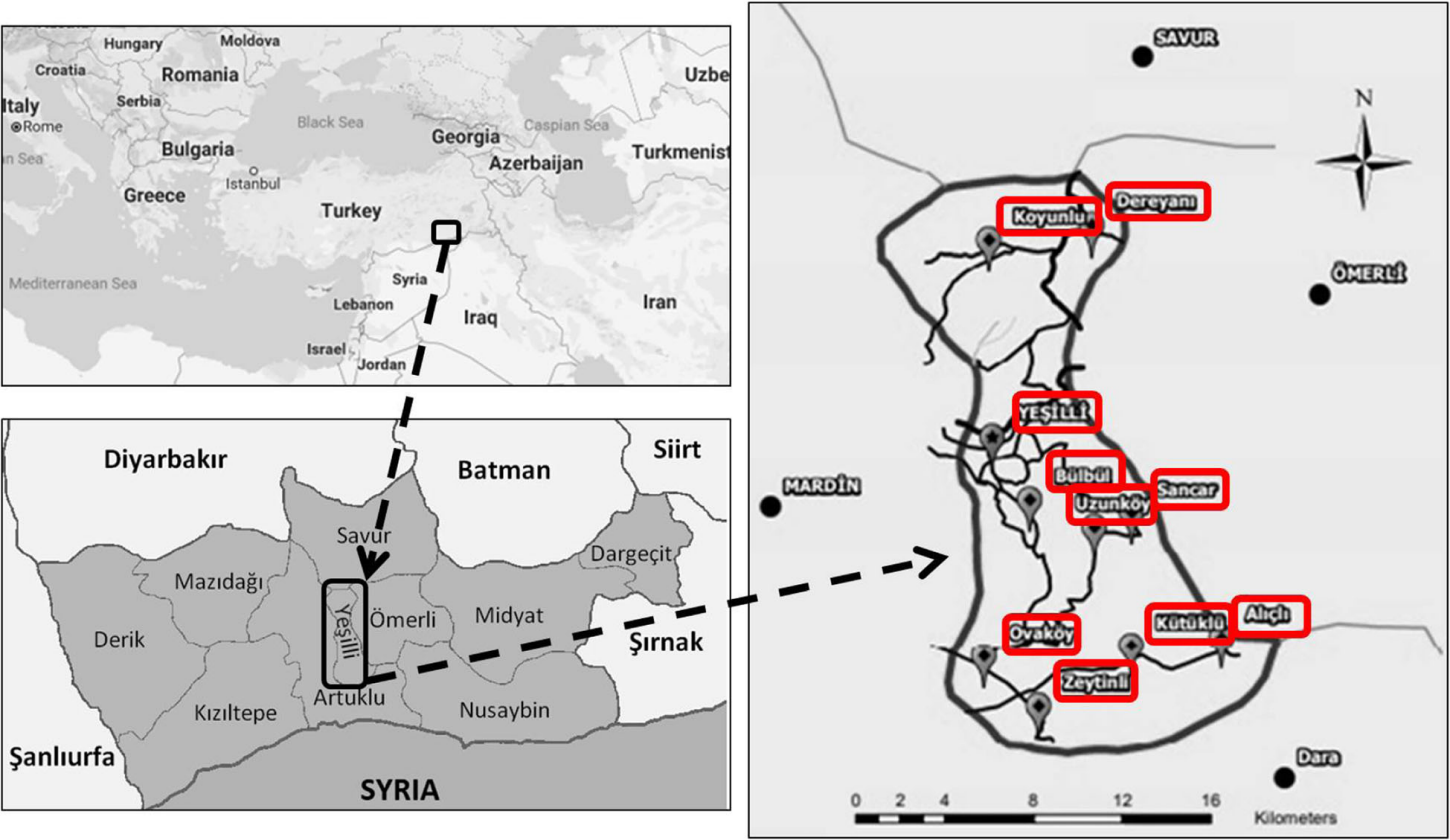
Map of Yeşilli and Districts. Provinces: Koyunlu, Dereyanı, Yeşilli city center, Bülbül, Sancar, Uzunköy, Alıçlı, Kütüklü, Ovaköy and Zeytinli

Yeşilli (formerly Rişmil) is a district of Mardin city, which is located in the southeast region of Turkey (37° 20′ 20.95′′ N and 40° 49′ 20.81′′ E) (Fig. 1). This area lies at an altitude of approximately 2696 ft a.s.l. Yeşilli district consists of nine rural settlements and five urban settlements.

Yeşilli has a transitional climate between the continental climate and the Mediterranean climate. The summers are hot, and the winters are cold. Most rainfall occurs in winter and spring [102]. The highest annual temperature is 38.5 °C (August), and the lowest is 0 °C (January) [103]. This area belongs to the Irano–Turanian Plant Geography Region and falls within the B8 grid square according to the grid classification system developed by Davis [13]. The area is covered with small oak forests and maquis. *Quercus coccifera* L., *Q*. *brantii* Lindl., *Olea europaea* Lindl., *Pistacia terebinthus* L., *Paliurus spina-christii* P. Mill. and *Juniperus oxycedrus* L. are among the important maquis species in this area [17]. The district is the greenest area in Mardin city.

### Socio-economic profile

**Fig. 2:**
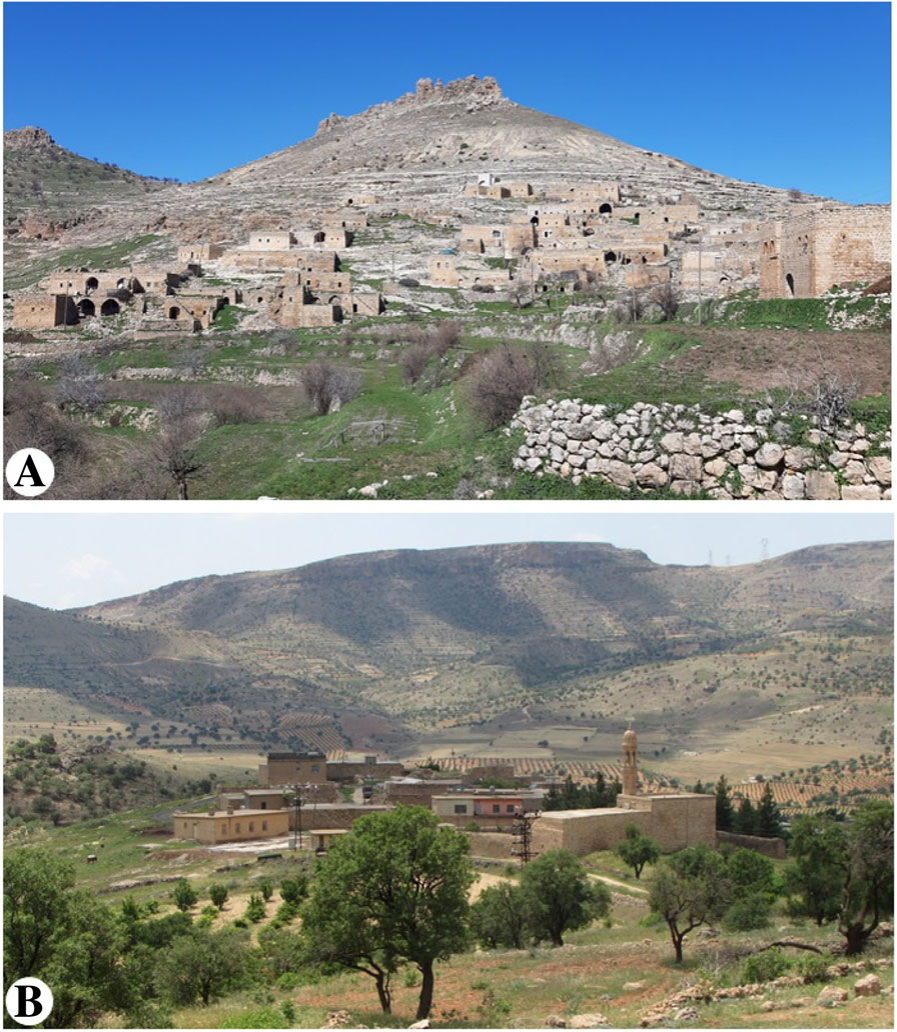
The villages of Yeşilli. **a** Sancar village. **b** Bülbül village

Yeşilli district is so named because horticulture and agriculture are highly developed in this region; ‘Yeşilli’ means ‘extensive greenery’. Almond and olive gardens and vineyards can be found in Bülbül, Dereyanı, Sancar and Uzunköy, as well as several other villages (Fig. 2). In other villages, sheep and goat breeding is common. People who live in Bülbül village prepare wine and olive oil to sell and trade almonds, olives and grapes. The inhabitants of other villages trade almonds, olives and grapes. In terms of agriculture, wheat, barley, lentils and chickpeas are cultivated.

Christians and Muslims have lived together for thousands of years in Yeşilli. Furthermore, four different languages, Turkish (official language), Kurdish, Arabic and Syriac, are spoken in this region. The population of this region is 16877 according to the address-based census of 2013 [102]. Yeşilli district has one Christian village, Bülbül. The inhabitants of this village are Assyrians, but they have forgotten their language; only those who study in the Monastery of Deyrulzafaran (Mardin) can write, read and speak the language. Twenty percent of the population can speak Syriac, and the older members of the population cannot speak Turkish at all. People speak Arabic, Kurdish and Syriac in their daily lives. Kurdish people live in the other eight villages, and they speak Kurdish, Turkish and sometimes Arabic. The majority of the population in the district centre is Arabic and speaks Arabic, Turkish and Kurdish.

## Methodology

### Survey and data collection

An ethnobotanical study was carried out between March 2017 and March 2019 to collect the knowledge of wild edible plant species being used by the local people in Yeşilli district and the surrounding areas. During this time, 120 plant specimens were collected. The collected plants were identified by the authors using ‘Flora of Turkey’ [13], ‘A Checklist of the Flora of Turkey (Vascular Plants)’ [16], ‘Illustrated Flora of Turkey Vol 1’ [17] and ‘Illustrated Flora of Turkey Vol 2’ [18]. Voucher specimens were deposited at the Herbarium of Faculty of Pharmacy, University of Istanbul (ISTE). The scientific names of the plant taxa were identified according to ‘A Checklist of the Flora of Turkey (Vascular Plants)’ [16] and The Plant List [104].

### Interviews with native people

We conducted interviews with local people without much difficulty because two authors (B. Yılmaz and M. Çelik) are local to the area and have relations there. The local community was informed, and their permission was received before conducting the questionnaire. We received guidance such as from teachers, imams (ministers) of mosques or headmen of the villages. A questionnaire was administered to the local people through face-to-face interviews. Interviews were conducted in the fields, houses and common areas of the villages. We visited the fields during all seasons and visited all villages at least twice. Elderly people who possessed deep knowledge of plants were visited at least three times, and sometimes we spent 1 or 2 days with these people in their farms, gardens, natural areas or houses. The International Society of Ethnobiology Code of Ethics was taken into account in interviews [105].

### Data analysis

The vegetable use category was further subcategorised as raw (Veg_R_), cooked (Veg_C_) or preserved (Veg_P_). Vegetables that are consumed after cooking, roasting or boiling, and those mixed with yogurt were categorised as cooked. Vegetables that are consumed after drying, adding to cheese or pickling were classified as preserved, and those consumed directly after washing, peeling or as salad were designated as raw.

Fruit usage was subcategorised as raw (Fr_R_) or processed (Fr_P_). Fruits that are eaten raw were categorised as raw, and processed fruits that are used after powdering, obtaining oil and drying were categorised as processed. Other categories included beverages (Bv), nectar (Ne), spices (Sp), latex (La) and medicinal plants (Med).

The information gathered through questionnaires and interviews was analysed quantitatively using two ethnobotanical indices as follows.

The factor informant consensus (*F*_*ic*_) was computed for each category to determine the homogeneity of the knowledge provided by the informants [105]. The Fic is calculated as follows:

$$ Fic=\frac{N_{ur}- Nt}{\ {N}_{ur}-1} $$

where *N*_ur_ refers to the number of use reports from informants for a particular use category, and *N*_t_ refers to the number of taxa that are used for a particular use category by all informants.

The cultural importance (CI) index was calculated for each species using the following formula [105]:

$$ \mathrm{CIs}=\Big[\sum \limits_{u^{=}{\mathrm{u}}_1}^{n_{\mathrm{N}\mathrm{C}}}{\sum}_{i={i}_1}^{{\mathrm{n}}_{\mathrm{N}}} UR\frac{ui}{N} $$]

The CI index can also be seen as the sum of the proportion of informants that mention the use of each species. This additive index takes into account not only the spread of the use (number of informants) for each species but also its versatility, i.e. the diversity of its uses. The theoretical maximum value of the index is the total number of different use categories (NC), which is reached in the unlikely case that all informants mention the use of the species in all use categories considered in a survey [105].

Jaccard’s similarity index considers the similarity between two OTUs (operational taxonomic units) as the number of attributes shared divided by the total number of attributes present in both OTUs. Jaccard’s index may be expressed as follows

$$ \mathrm{J}=\mathrm{C}/\mathrm{A}+\mathrm{B} $$

Where *A* is the number of attributes present in OTU a, *B* is the number of attributes present in OTU b and *C* is the number of attributes present in both OTUs a and b. The number of attributes present in either of the OTUs is given by *A* + *B* [106].

## Results and discussion

### Socio-economic characteristics of informants

A total of 62.9% female and 37.1% male informants were interviewed. The informants had varying levels of education, with 33.87% having no education, 46.77% having a primary level, 14.51% having a secondary level and only 4.83% having a tertiary level. The demographic details of the informants can be seen in Table 1.

**Table 1:** Demographic detail of the interviewed informants

Category	Subcategory	% of informants
Gender	Female	62.9
	Male	37.1
Age	15-40	7
	40-60	31
	60 and older	24
Education level	None	33.87
	Primary	46.77
	Secondary	14.51
	Tertiary	4.83

### Diversity of wild edible plant taxa

In multicultural regions, differences in the use of wild vegetables could be interpreted as a result of complex interactions between cultural preferences [99]. The use of plants varies in the present study area. The present study found 74 plant taxa belonging to 31 plant families that are used as wild edible plants in Yeşilli district (Mardin). Table 2 lists details of the utilised wild edible plant species arranged in alphabetical order, including scientific name, family name, herbarium number, local name (Arabic, A; Syriac, S; and Kurdish, K), life form, plant parts used, modes of consumption, CI and previous ethnobotanical literature records from Turkey and bordering countries. The results show that most of the plants used are Asteraceae (ten taxa), followed by Rosaceae (seven taxa), Fabaceae (six taxa), Lamiaceae (four taxa), Brassicaceae (four taxa), Polygonaceae (four taxa) and Boraginaceae (three taxa). The remaining plant families are represented by only one or two species. Because of the dominance of the Asteraceae, Rosaceae and Fabaceae families in the flora of the studied area, plants from these families are used extensively. These results are in general agreement with those of previous studies [2, 29, 40, 81, 99]. However, some of previous studies [60, 70, 82] reported that most of the wild edible plants were in the Apiaceae family. While others [68] reported that Fabaceae is the most important family of wild edible plants. The use of plants from different families indicates the intensity and importance of this information. These results are in accordance with those found in studies conducted in Turkey [31, 32, 60, 70, 81, 82] and bordering countries [97–99]. However, in some studies conducted in other parts of the world, the most commonly used plants are in Rosaceae family [1–4, 108, 109].

**Table 2:** List of wild plants used as foodstufs in Yeşilli District

Scientific name Family name	Voucher number (ISTE)	Local name (Languages; A, K, S)	Ethnic groups (Ar, As, Ku) (Number of interviewes)	Life form	Plant part used	Modes of consumption	CI	Previous ethnobotanical literature records
*Allium ampeloprasum* L. Amarylidaceae	115232	Summe (A), Sirik (K)	As (1), Ku (18)	Herb	Leaves, Bulbs	Boiled then added to yogurt and bulgur soup, added into cheese Raw as salad	0.31	[44, 52, 60, 70, 81]
*Allium chloranthum* Boiss. Amarylidaceae	115738	Serspik Sirik, Sirim (K)	Ku (10)	Herb	Leaves	Added into cheese, raw, added to salad	0.16	Not reported
*Allium wendelboanum*Kollmann^a^ Amarylidaceae	115 737	Serspik, Sirik, Sirim (K)	Ku (6)	Herb	Leaves	Added into cheese Raw as salad	0.09	Not reported
*Amaranthus retroflexus* L. Amaranthaceae	115734	Koksor (K)	Ku (25)	Herb	Aerial parts	Boiled then fried with egg, cooked as soup with yogurt and bulgur	0.40	[32, 33, 37, 40–42, 50, 51, 69, 80–82, 94]
*Anchusa strigosa* Banks & Sol. Boraginaceae	115247	Hımhım (A), Gûrîz (K)	Ar (11), As (5), Ku (39)	Herb	Young leaves Flowers nectar	Boiled then fried with onion, medicinal Its nectar sucked	0.88	[26, 31, 94]
*Arum rupicola* Boiss. Araceae	116014	Kardi (K)	Ar (3), Ku (10)	Herb	Leaves	Boiled then fried with onion, medicinal	0.21	[55, 70, 72, 94, 98]
*Capparis sicula* subsp. *sicula* Veill. Capparaceae	115267	Kebere (K)	Ar (2), As (4), Ku (14)	Shrub	Buds	Raw as salad, pickled	0.32	[26–28, 41, 52, 56, 65]
*Celtis tournefortii* Lam. Cannabaceae	115293	Gernoso (S) Gıngırêz (A) Teêv, Tuêv (K)	As (5), Ku (20)	Tree	Fruits	Raw as a snack, prepared a mixture, medicinal	0.40	[28, 40, 49, 81, 82]
*Centaurea hyalolepis* Boiss. Asteraceae	115257	Sitrîzerk(K)	Ku (9)	Herb	Young leaves	Raw as salad	0.14	[26]
*Centaurea virgata* Lam. Asteraceae	115268	Deqnişeh (A), Tal (K)	As (2)	Herb	Young leaves	Raw as salad	0.03	Not reported
*Cerasus mahaleb* var. *mahaleb* (L.) Mill. Rosaceae	115684	Mahlab (S) Mahlep, Mahleb (A-K)	As (5) As (5), Ku (4)	Shrub	Fruits	Prepared liqueur and wine Raw as a snack, medicinal	0.08 0.15	[70]
*Cerasus microcarpa* subsp. *microcarpa* (C.A.Mey.) Boiss. Rosaceae	115677	Fıkt (A)	As (3), Ku (26)	Shrub	Fruits	Raw as a snack, medicinal	0.29	[49, 60, 94]
*Cerasus prostrata* var. *prostrata* (Labill.) Ser. Rosaceae	115732	Belalûk (K) Fıkt (A) Ĥorişmê (S)	Ku (11)	Shrub	Fruits	Raw as a snack, medicinal	0.18	Not reported
*Chenopodium album* L. Amaranthaceae	115729	Serbîmast (K)	Ku (12)	Herb	Young aerial parts	Boiled and then fried eggs added, cooked a soup with yogurt and bulgur	0.19	[30, 32–34, 37, 38, 42, 43, 49, 53, 54, 59, 63, 65, 69, 71, 72, 78–82, 84, 88, 89, 92–94]
*Chondrilla juncea* var. *juncea* L. Asteraceae	115248	Ilke (A)	Ku (9)	Herb	Young leaves	Raw as salad, added to salad	0.14	[33, 38, 41, 42, 50, 65, 78–80, 94]
*Convolvulus arvensis* L. Convolvulaceae	115674	Lavlavk (K), Lıblebê (A)	As (3), Ku (16)	Herb	Aerial parts	Boiled and then fried eggs added, cooked a soup with yogurt and bulgur	0.19	[27, 32, 37, 58, 59, 63, 65, 74, 75, 78, 89, 92, 94]
*Crataegus azarolus* var. *azarolus* L. Rosaceae	115250	Givîj, Gîjok, Gûhîj (K), Ğızran (A)	As (5), Ku (33)	Tree	Fruits	Raw as a snack	0.61	[52, 82, 96]
*Crocus cancellatus* Herbert subsp. *damacenus* (Herbert) Mathew Iridaceae	115940	Hılhılêye (A) Pîvok (K)	As (3), Ku (35)	Herb	Corm	Raw as a snack after pelling off the outher part	0.61	[26–28, 79, 94]
*Echinophora tenuifolia* L. subsp. *sibthorpiana* (Guss.) Tutin Apiaceae	115670	Mırbela (A)	As (5)	Herb	Young stems	Raw as a snack after peeling off the outher part	0.08	[26, 27, 41–43, 49, 58, 74, 78, 81, 92]
*Echinops orientalis* Trautv. Asteraceae	115703	Gelo helvo pevero (S)	As (5) Ku (21)	Herb	Receptacle Dried latex	Raw as a snack	0.16	[27, 40, 60, 70, 78]
*Echinops spinosissimus* Turra subsp. *bithynicus* (Boiss.) Greuter Asteraceae	115283	Gelo helvo pevero (S)	As (5)	Herb	Receptacle Dried latex	Raw as a snack	0.08	[40]
*Echium italicum* L. Boraginaceeae	115234	Hımhım (A), Mijok (K)	As (2), Ku (9)	Herb	Flowers, nectar	Its nectar sucked, medicinal	0.18	[26, 40–42]
*Elaeagnus angustifolia* L. Elaeagnaceae	115235	Sinç (K)	Ku (31)	Shrub	Fruits Flowers	Raw as a snack As tea	0.50	[32, 36, 37, 49, 52, 69, 78, 79, 93, 94]
*Erodium cicutarium* (L.) L’Hér Geraniaceae	116438	Derzikepire (K)	Ar (5), Ku (15)	Herb	Leaves, fruits, stems	Eaten raw Raw as a snack after pelling off the outher part	0.32	[26, 31, 41, 42, 50, 57, 58, 65, 73, 75, 89, 99]
*Eryngium creticum* Lam. Apiaceae	115236	Beektire/Ekkeyde (A)	Ar (15), As (4)	Herb	Petioles Young stem, young roots	Raw as a snack Raw as a snack after pelling off the outher part, medicinal	0.29	[48, 49, 73]
*Euphorbia helioscopia* subsp. *helioscopia* L. Euphorbiaceae	115254	Lahye (A)	As (2), Ku (8)	Herb	Aerial parts	Added to molasses, to improve the test of molasses, direct or dry	0.16	[41]
^b^*Ficus carica* subsp. *carica* L. Moraceae	115943	Hejîr (K) Tin (A)	Ar (15), As (5), Ku (39)	Tree	Fruits	Raw as a snack, dried eaten	0.96	[20, 26, 37, 38, 40, 43, 50, 52, 55, 56, 58, 59, 62, 63, 72, 75, 82, 86, 92, 96]
*Ficus carica* L. subsp. *rupestris* (Hausskn.) Browicz Moraceae	115699	Kerik hejîr (K), Tin (A)	As (5), Ku (15)	Shrub	Fruits	Raw as a snack, dried eaten	0.32	[27, 40, 94]
*Gagea villosa* (M.Bieb.) Sweet Liliaceae	116418	Şeqılarab(A)Şînêrak(K)	Ku (10)	Herb	Corm	Raw as a snack after peeling off the outher part	0.16	Not reported
*Geocaryum cynapioides* (Guss.) Engstrand subsp. *macrocarpum* (Boiss. & Spruner) Menemen Apiaceae	115681	Ceviz il arz (A) Gûzê ardê (K)	As (5)	Herb	Tubers	Raw as a snack	0.08	Not reported
*Glycyrrhiza glabra* L. Fabaceae	115 667	Meyan/Sus/ Sûs (A-K)	Ar (12), As (5), Ku (34)	Herb	Roots	Prepared as syrup, medicinal	0.83	[26, 27, 32, 41, 48, 52, 78, 79, 81, 82, 93]
*Gundelia tournefortii* L. Asteraceae	115711	Arkue (A), Êrgudê (S) Kereng, Herşev (K),	Ar (6), As (5), Ku (39)	Herb	Petioles Roots, stems	Raw as a snack Young stems and roots are eaten after pelling off the outher part as a snack, boiled and then fried, eggs added, boiled and then the soup is preparedby adding yogurt and bulgur	0.80	[26, 27, 31, 33, 40–42, 52, 55, 60, 65, 70, 72, 78, 80–82, 84, 86, 89, 94, 96, 98, 99]
*Hirschfeldia incana* (L.) Lagr.-Foss. Brassicaceae	115307	Herdlo (S) Xerdel (A-K)	Ku (7)	Herb	Young aerial parts	Raw as a snack, as salad, medicinal	0.11	[107]
*Iris persica* L. Iridaceae	116013	Birbîzêk, Birbîzêka mîha (K), Birxızeylê (A)	As (2) Ku (8)	Herb	Tepals	Raw as a snack	0.16	[82, 94]
*Iris reticulata* M. Bieb. Iridaceae	116000	Birbîzêk, Birbîzêka bizina (K), Birxızeylê (A)	As (2), Ku (8)	Herb	Tepals	Raw as a snack	0.16	[31, 82]
^b^*Juglans regia* L. Juglandaceae	115679	Cevz (A) Gûz (K)	Ar (12), As (5), Ku (28)	Tree	Seeds	Eaten as dried nuts	0.73	[20, 28, 35–38, 40, 41, 49, 55, 57, 60, 62, 64, 66, 68, 70–72, 82, 92, 94]
*Lathyrus cassius* Boiss. Fabaceae	115721	Baqilê bizinan (K)	Ku (10)	Herb	Young seeds	Raw as a snack	0.16	Not reported
*Lepidium draba* L. Brassicaceae	115252	Qınêber (K)	Ar (18), As (5), Ku (37)	Herb	Young aerial parts	Raw as a snack, as salad, fried with salad and eggs added	0.96	[26, 27, 31, 32, 42, 44, 53, 55, 69, 81, 84, 89, 93]
*Malva neglecta* Wallr. Malvaceae	115242	Hıbbeze (A), Tolig, Tolik (K)	Ar (10), As (4), Ku (34) Ku (30)	Herb	Young aerial parts Fruit	Boiled and then fried with onion, medicinal Raw as a snack	0.77	[26, 28, 30–34, 36, 38, 40, 44, 47–49, 51–55, 59, 62, 65, 76, 78–82, 84, 88, 89, 94, 98, 99]
*Mentha longifolia* (L.) L. Lamiaceae	115243	Nahne, Nınha (A), Pûng (K)	Ar (18), As (3), Ku (26)	Herb	Leaves, young aerial parts	As spices, medicinal As tea	0.75	[32–34, 38, 40, 44, 47–49, 51–54, 58, 60, 62, 63, 65, 69, 70, 72, 74–76, 78, 80–82, 84–89, 94, 96, 98, 99]
*Nasturtium officinale* R.Br., Aiton Brassicaceae	115903	Tûzik (K)	Ku (31)	Herb	Young aerial part	Raw as a snack, as salad	0.50	[41–43, 51, 55, 59, 64, 71, 74, 76, 78, 80, 82, 98, 99]
*Notobasis syriaca* (L.) Cass. Asteraceae	116415	Aynbeloqê (A) Kelbeşe belek (K)	Ar (7), As (4), Ku (39)	Herb	Young stems	Raw as a snack after pelling off the outher part	0.80	[26, 28, 50, 96]
^b^*Olea europaea* L. Oleaceae	115262	Zeitûn (A-K)	Ar (8), As (5), Ku (20)	Tree	Fruit, oil	Eaten conserved, medicinal	0.50	[37, 38, 42, 50, 71, 92]
*Onopordum carduchorum* Bornm. & Beauverd Asteraceae	116442	Kelbeşakaran (K)	Ar (6), As (5), Ku (39)	Herb	Young stems	Raw as a snack after pelling off the outher part	0.80	[28, 31, 55]
*Onosma roussaei* DC. Boraginaceae	115712	Hımhım (A), Mîjok (K)	Ku (7)	Herb	Flowers nectar	Its nectar sucked	0.12	[31]
*Onosma alborosea* Fisch. & C.A.Mey. Boraginaceae	115316	Hımhım (A), Mîjok (K)	As (2), Ku (10)	Herb	Flowers nectar	Its nectar sucked	0.19	Not reported
*Papaver glaucum* Boiss. & Hausskn. Ex Boiss. Papaveraceae	115249	Kulîlka Bûk û Zava, Kulîlka Nisanê, Kulîlka erebo (K), Qırçeh (A)	Ku (12)	Herb	Young leaves	Raw as salad, fried, medicinal	0.19	Not reported
*Papaver macrostomum* Boiss. & A.Huet Papaveraceae	115680	Kulîlka Nisanê (K)	As (3), Ku (32)	Herb	Young leaves	Raw as salad, fried, medicinal.	0.22	[65, 78, 94, 99]
*Pistacia terebinthus* L. subsp. *palaestina* (Boiss.) Engler Anacardiaceae	115944	Batmê (S) Bittim (K) Bıttım (A)	Ar (10), As (4), Ku (16)	Shrub	Unripe fruits	Raw as a snack or preparing a coffee	0.48	[30, 38, 41–43, 50, 52, 56, 59, 60, 65, 75, 78, 81, 86]
*Pisum fulvum* Sibth. & Sm. Fabaceae	115724	Baqilê xatûnî (K)	Ku (10)	Herb	Young seeds	Raw as a snack	0.16	Not reported
*Polygonum cognatum* Meissn. Polygonaceae	115275	Casûrik (K)	Ar (5)	Herb	Young aerial parts	Raw as a snack, as salad, fried or boiled then added to yogurt	0.08	[32–36, 40–44, 48, 49, 52, 54, 55, 58, 59, 65, 70, 75, 76, 78, 80, 81, 84, 88, 89, 93, 94]
*Portulaca oleracea* L. Portulacaceae	115241	Pirpar (A-K), Parparık (K)	As (5), Ku (19)	Herb	Aerial parts	Raw in salads, cooked as vegetable, medicinal.	0.38	[31, 33, 55, 59, 70, 75, 82, 96, 98]
*Quercus brantii* Lindl. Fagaceae	115902	Berû (K)	As (1), Ku (17)	Tree	Seed	Roasted in embers, medicinal	0.29	[31, 38, 52]
*Rosa canina* L. Rosaceae	115255	Gulşîlav (K)	As (5), Ku (28)	Shrub	Fruits	Raw as a snack, medicinal	0.53	[20, 32–36, 38, 40, 41, 47–49, 51–53, 55, 59, 60, 62, 63, 65, 66, 69–72, 74, 75, 78, 81, 82, 86, 93, 94, 96]
*Rosa foetida* J. Herrm. Rosaceae	116444	Verdasfar (A), Gulşîlave zer (K)	As (4), Ku (36)	Shrub	Petals	Raw as a snack	0.65	Not reported
*Rosularia radiciflora* Borris. Crassulaceae	116459	Tirîke pîrê (K) Tulfilasfor (A)	As (2), Ku (5)	Herb	Leaves	Raw as a snack	0.11	Not reported
*Rhus coriaria* L. Anacardiaceae	115280	Sımaq (A-K)	Ar (18), As (5), Ku (32)	Shrub	Fruit	As spice	0.88	[20, 28, 31, 34, 38, 41, 52, 55, 60, 65, 66, 71, 75, 78, 81, 82, 86, 92, 96]
*Rubus sanctus* Schreb. Rosaceae	115306	Durîreşk, Durîhişhişok (K)	As (3), Ku (30)	Shrub	Fruits	Raw as a snack	0.54	[20, 30, 31, 37, 38, 49, 50, 55, 60, 63, 66, 71, 75, 81, 82, 92]
*Rumex crispus* L. Polygonaceae	115271	Sumaksevaqi (A), Tirşuq (K)	As (3), Ku (9)	Herb	Young leaves	As a wrapping material for ‘sarma’	0.19	[32, 33, 36, 37, 41–43, 49, 52–55, 58, 65, 69, 71, 75, 76, 78, 80, 84, 88, 96, 98]
*Rumex obtusifolius* L. subps. *subalpinus* (Schur) Celak. Polygonaceae	115309	Sumaksevaqi (A), Sabûna leglegê (K)	As (3), Ku (12)	Herb	Young leaves	As a wrapping material for ‘sarma’	0.24	[41, 45, 75]
*Rumex tuberosus* L. subsp. *horizantalis* (Koch) Rech. Polygonaceae	115256	Sumaksevaqi (A), Tirşuq (K)	As (4), Ku (7)	Herb	Young aerial parts	Raw as a snack	0.18	[30, 32, 36, 44, 47, 53, 59, 60, 70, 72, 76, 94]
*Salvia syriaca* L. Lamiaceae	115671	Carboe (A)	Ar (14)	Herb	Gall	Raw as a snack	0.23	[31, 52, 94]
*Sedum pallidum* Bieb. Crassulaceae	116434	Tirîke pîrê (K) Tulfilasfor (A)	As (2), Ku (5)	Herb	Leaves	Raw as a snack	0.11	Not reported
*Silene dichotoma* Erh. Caryophyllaceae	116417	Şekirok (K)	As (5)	Herb	Young seeds	Raw as a snack	0.08	[31, 33, 55]
*Sinapis alba* subsp. *alba* L. Brassicaceae	115315	Xerdel (A-K)	Ar (18), As (4), Ku (29)	Herb	Young aerial parts Young leaves	Fried with onion Raw as a snack	0.82	[26, 28, 41, 42, 92, 99]
*Thymbra spicata* subsp. *spicata* L. Lamiaceae	115700	Cehter, Cahter (K)	As (4), Ku (25)	Shrub	Aerial parts	As spice	0.46	[28, 30, 31, 38, 43, 55, 74, 78, 86]
*Thymus kotschyanus* subsp. *kotschyanus* Boiss. & Hohen. Lamiaceae	115706	Cehter, Cahter (K)	Ku (9)	Shrub	Aerial parts	As spice	0.14	[32, 38, 60, 62, 70, 74, 78, 81, 82]
*Tragopogon latifolius* Boiss. var. *angustifolius* Boiss. Asteraceae	115687	Gezrik (K)	Ku (7)	Herb	Young leaves and roots	Raw as a snack	0.11	[33, 42, 58, 65, 89, 94]
*Tragopogon porrifolius* L. subsp. *longirostris* (Sch. Bip.) Greuter Asteraceae	116428	Gezrik (K)	Ku (6)	Herb	Young leaves and roots	Raw as a snack	0.10	[31, 41, 42, 99]
*Urtica dioica* L. Urticaceae	115253	Gezgezik (K) Kırays (A)	Ar (14), As (5), Ku (26)	Herb	Young aerial parts	Fried with onion and then eggs added, medicinal	0.72	[20, 26, 28, 30–34, 36, 40, 42–44, 48–50, 52–55, 58–60, 62–65, 69–72, 75–81, 86, 88, 89, 92, 94, 98]
*Urtica pilulifera* L. Urticaceae	115258	Gezgezok (K)	Ku (15)	Herb	Young aerial parts	Raw as a snack, fried with onion and then eggs added, medicinal	0.24	[30, 33, 36, 55]
*Vicia palaestina* Boiss. Fabaceae	115723	Baqilê çukan (K)	Ku (11)	Herb	Young seed	Raw as a snack	0.18	[26]
*Vicia pannonica* var. *pannonica* Crantz Fabaceae	115716	Baqilê gan (K)	Ku (10)	Herb	Young seed	Raw as a snack	0.16	[31]
*Vicia sativa* L. subsp. *nigra* (L.) Ehrh. var. *nigra* Fabaceae	115722	Baqilê maran (K)	Ku (18)	Herb	Young seed	Raw as a snack	0.29	[27, 49, 52, 63]

ISTE Herbarium of Faculty of Pharmacy, University of Istanbul, *A* Arabic, *Ar* Arab, *As* Assyrian, *K* Kurdish, *Ku* Kurd, *S* Syriac

^a^Endemic

^b^Both cultivated and wild plants

When comparing the results of the present study with those of ethnobotanical studies in Turkey, the regions in which plants and their use most overlap are as follows, listed according to the number of taxa: Midyat (Mardin) (23 taxa) [31], Malatya (22 taxa) [94], Bingöl (19 taxa) [82], Cizre (Şırnak) (18 taxa) [52], Elazığ (17 taxa) [81], Urfa (16 taxa) [26], Tunceli (15 taxa) [40] and Hakkari (13 taxa) [60]. Since the region where this present study was conducted is close to Midyat [31] and has the same cultural structure and geography as Mardin, some of the plants and their uses overlap with those identified in the Midyat study, as were expected. Other areas where overlaps are intense are Turkey’s southeast (including Yeşilli), and the Eastern Anatolia Region. These regions are located in the same phytogeographic region and have almost the same cultural structure. Also, overlaps were observed with ethnobotanical studies conducted in bordering countries with the same cultural structure: Iraq (11 taxa) [99], southern Iraq (9 taxa) [96], Hawraman (Iraq) (8 taxa) [98] and Armenia (8 taxa) [97].

Additionally, when our results were compared with those of an ethnobotanical study conducted in Midyat, close to Yeşilli, we observed many similarities between the local names of plants because of the similar cultural structures areas. The local names of 14 taxa are similar: *Cerasus mahaleb* var. *mahaleb*, *Convolvulus arvensis*, *Erodium cicutarium*, *Gundelia tourneforti*, *Malva neglecta*, *Pistacia terebinthus* subp. *palaestina*, *Rhus coriaria*, *Thymbra spicata* and *Urtica dioica*. Also, when we compared the current study with an ethnobotanical study conducted in Cizre (Şırnak), we observed similarities between the local names of 13 taxa: *Crataegus azarolus* var. *azarolus*, *Elaeagnus angustifolia*, *Ficus carica* subsp. *carica*, *Glycyrrhiza glabra*, *Gundelia tourneforti*, *Malva neglecta*, *Mentha longifolia*, *Pistacia terebinthus* subp. *palaestina*, *Rhus coriaria*, *Quercus brantii*, *Rhus coriaria*, *Rumex crispus* and *Urtica dioica*. It is already known that the common language of these areas is Kurdish.

We further observed many similarities between the local names of plants from Yeşilli and those from bordering countries: Armenia (six taxa) [97], Hawraman (Iraq) (six taxa) [98] and Iraq (four taxa) [99].

Furthermore, almost 12 taxa (c.16%) were also available at the public market and local shops. This shows that the plants are largely freshly harvested and consumed and the persistence and possibly economic viability of the domestic, small-scale wild vegetable trade.

### Differences among the three studied communities

**Fig. 3:**
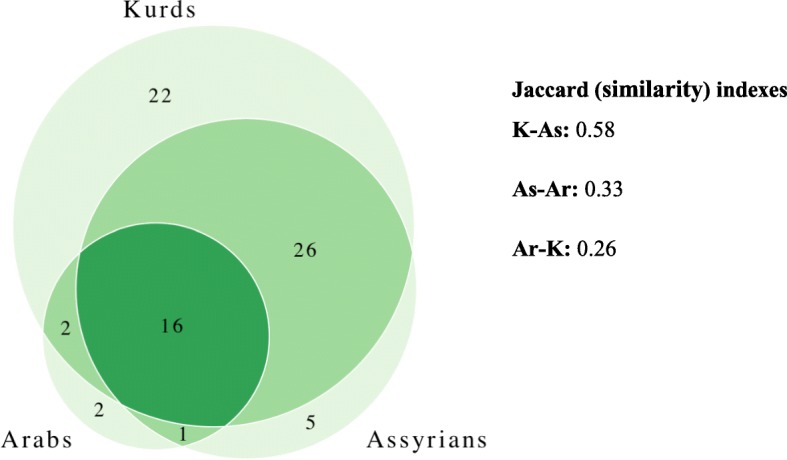
Overlap in the wild edible plants traditionally gathered by three studied communities

In this study, the number of interviewed Kurd informants was 39, Arabs was 18 and Assyrians was five. The consumption ratio of wild edible plants is as follows: Kurds (80.26%), Assyrians (60.52%) and Arabs (23.68%). The most overlap of the obtained data and the Jaccard index (similarity) was between Kurds and Assyrians and the least one was between Arabs and Kurds, perhaps due to the different main ecological areas where wild edible plants were collected (Fig. 3). The Kurds, whose livelihood is horticulture and mainly husbandry, have maintained gathering wild edible plants around their villages and from high mountains for a long period of time. In Bülbül village (the Assyrian village), since people generally speak in Arabic, the Syriac names of the plants had mostly forgotten. Therefore, most of the plants’ names were recorded in Arabic. Although fewer people were interviewed in the Assyrian village due to the law population, the use of many plants clearly indicates that they continue to use wild edible plants extensively and continue to hand down this traditional knowledge to the next generations. Assyrian beliefs have also an important role on consuming the plants. For instance, an informant who was educated in the Darülzefaran Monastery (an Assyrian Monastery) stated that parts with in the form of a cross are harmless and can be eaten or used. On the other hand, although more Arabs were interviewed than the Assyrians, less traditional knowledge was recorded. The reason for this is that because the Arabs live in the district center where there is higher capability to buy food. They often consume the plants that the Kurds gather from their villages and surrounding mountains and bring them to the public markets and local shops.

The most culturally important taxa were *Ficus carica* subsp. *carica*, *Lepidium draba*, *Anchusa strigosa*, *Rhus coriaria*, *Glycyrrhiza glabra*, *Sinapis alba*, *Gundelia tournefortii*, *Notobasis syriaca*, *Onopordum carduchorum*, *Malva neglecta*, *Mentha longifolia*, *Juglans regia* and *Urtica dioica*. However, these plants are popular among the three communities in different manners. In addition to these plants *Olea europaea*, *Pistacia terebinthus* subsp. *palaestina* and *Capparis sicula* which has low cultural index are also consumed by three communities. However, *Anchusa strigosa*, *Gundelia tournefortii*, *Notobasis syriaca* and *Onopordum carduchorum* were mainly mentioned by Assyrians and Kurds. While *Rosa canina*, *R*. *foetida*, *Rubus sanctus*, *Crataegus azarolus* var. *azarolus*, *Crocus cancellatus* subsp. *damacenus* and *Thymbra spicata* subsp. *spicata* with high cultural index prevailed only by Assyrians and Kurds, *Arum rupicola* and *Erodium cicutarium* were mentioned by Arabs and Kurds and *Eryngium creticum* were mentioned by Arabs and Assyrians.

On the other hand, *Centaurea virgata*, *Echinophora tenuifolia* subsp. *sibthorpiana*, *Echinops spinosissimus* subsp. *bithynicus*, *Geocaryum cynapioides* subsp. *macrocarpum* and *Silene dichotoma* were only mentioned by Assyrians and *Salvia syriaca* and *Polygonum cognatum* were only mentioned by Arabs. Twenty-two taxa were mentioned only by Kurds, among these species *Amaranthus retroflexus*, *Elaeagnus angustifolia*, *Nasturtium officinale* and *Vicia sativa* subsp. *nigra* were metioned particularly by many interviewees.

Producing herbal cheese is common among the Kurds living in high altitude villages, similar to the eastern and the southeast regions of Turkey [74]. The beverage called ‘ava susê’, prepared from roots of *Glycyrrhiza glabra* is also common among them. However, only the Assyrians use the fruits of *Cerasus mahaleb* var. *mahaleb* to produce liqueur and wine. Besides, it was recorded that the leaves of *Arum rupicola* were consumed by Arabs and Kurds.

### Informant consensus index (F_ic_)

The informant consensus index (*F*_*ic*_) varied between 0.99 for preserved vegetables and 0.95 for raw vegetables (Table 3). The *F*_*ic*_ for beverages and spices was 0.98_._ These results reveal that although different cultures and different environments are present in the study area, people continue to use wild food plants intensively.

**Table 3:** Wild edible plant taxa and their cultural importance of various use-categories and subcategories

Use-category/subcategory	Number of species	The number of use reports (UR)	CI	*Fic*
Vegetables	46	1011	16.30	0.96
Vegetable (raw)	35	670	10.81	0.95
Vegetable (cooked)	20	443	7.14	0.96
Vegetable (preserved)	6	102	1.65	0.95
Fruits	14	391	5.77	0.97
Fruit (raw)	14	384	6.19	0.97
Fruit (processed)	3	176	2.83	0.99
Seeds	8	127	2.04	0.94
Seed (raw)	7	109	1.75	0.94
Seed (cooked)	2	53	0.85	0.98
Beverage	5	168	2.71	0.98
Nectar	3	74	1.19	0.97
Spices	4	140	2.25	0.98
Latex	2	10	0.16	0.89
Medicinal WEPs	17	480	7.74	0.96

### Use categories and cultural importance of wild edible plants

**Fig. 4:**
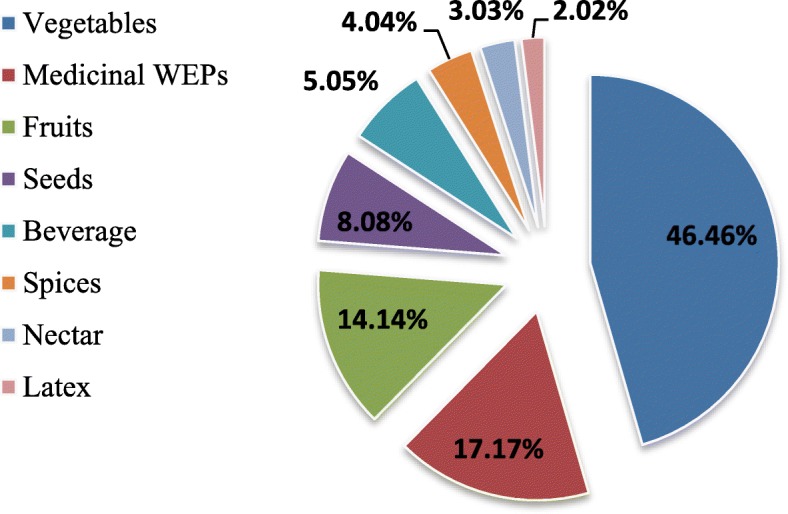
Use categories and the percentage of plants that fall into each category according to the data of the present study

**Fig. 5:**
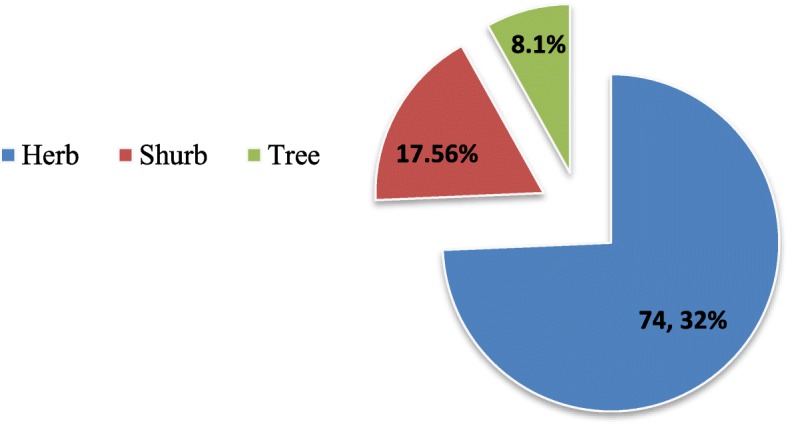
Life forms of wild edible plants

We classified the wild edible plants into eight categories. The category with the most plants was vegetables (46 taxa), followed by medicinal plants (17 taxa) and fruits (14 taxa) (Fig. 4). The CI index value of vegetables was 42.71% of the total CI, followed by medicinal WEPs 20.28%, fruits 15.12% and beverages 7.10%. The maximum number of use reports was recorded for vegetables (1011) (Table 3). The life forms of wild edible plant taxa include mainly herbs (74.32%, 55 taxa), followed by shrubs (17.56%, 13 taxa) and trees (8.1%, 6 taxa) (Fig. 5). According to the CI index, the most important vegetable taxa were *Lepidium draba* (CI 0.96), *Anchusa strigosa* (CI: 0.88), *Sinapis alba* (CI: 0.83), *Gundelia tournefortii* (CI: 0.80), *Notobasis syriaca* (CI: 0.80), *Onopordum carduchorum* (CI: 0.80), *Malva neglecta* (CI: 0.77), *Urtica dioica* (CI: 0.72), *Rosa foetida* (CI: 0.65), *Crocus cancellatus* subsp. *damascenus* (CI: 0.61), *Nasturtium officinale* (CI: 0.50) and *Amaranthus retroflexus* (CI: 0.40). The most common fruit consumed by the locals were *Ficus carica* subsp. *carica* (CI: 0.96), *Crataegus azarolus* var. *azarolus* (CI: 0.61) *Rubus sanctus* (CI: 0.54), *Rosa canina* (CI: 0.53) and *Pistacia terebinthus* subsp. *palaestina* (CI: 0.48).

### Vegetables

The intense, diverse use of wild vegetables was reported in East Turkey and near the border of Turkey [99]. The present study confirms previous results because most taxa with high CI index value were vegetables. While *Sinapis alba* is a culturally important species in the study area, its usage is not very common in Turkey [26, 28, 41, 42, 92, 99]. *Malva neglecta* has substantial local value as a vegetable in Turkey and bordering countries [26, 28, 30–34, 36, 38, 40, 44, 47–49, 51–55, 59, 62, 65, 76, 78–82, 84, 88, 89, 94, 98]. Also it is a necessary plant in Yeşilli; the young aerial parts of *Malva neglecta* are boiled and then fried with onion. It is rich in vitamins A, B and C [110].

**Fig. 6:**
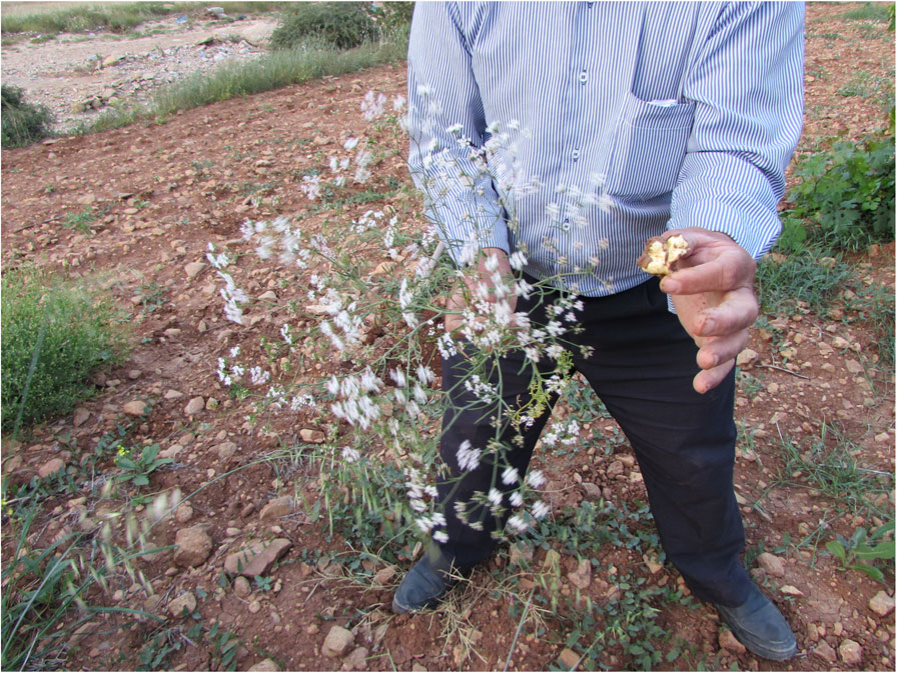
*Geocaryum cynapioides* and its tuber

The tubers of *Geocaryum cynapioides* are peeled and eaten raw (Fig. 6). This plant is thought to have a brain-like appearance and is believed to be a tonic for the brain when consumed. The corms of *Crocus cancellatus* subsp. *damascena* and *Gagea villosa* are collected in early spring, then corm tunics are peeled and the fleshy part is eaten raw. The same use of *Crocus cancellatus* subsp. *damascenus* was previously reported in the southeast and eastern region of Turkey [26–28, 79, 94]. *Gagea villosa* is widely distributed in Turkey; however, the use of it has never been repoted to be used as. While the gall of *Salvia syriaca* is eaten raw in Yeşilli, as consumed in Kürecik [94], its fruits are consumed as spices in Midyat [31], flowers as herbal tea in Cizre [79].

The consumption of *Centaurea hyalolepis* leaves was recorded in the present study for first time. A previous study [26] reported that the flowers of the plant were eaten. Additionally, the consumption of *Centaurea virgata* as food was also recorded only in this study; this plant is named ‘tal’, which means ‘hot’. The reason for this taste is the chemical compounds of the species, including sesquiterpenes, flavones and the flavonol isokaempferide [111].

Cooked plants are fried and then consumed or boiled and then a soup is prepared by adding yogurt and boiled bulgur. For instance, the aerial parts of *Gundelia tournefortii*, *Lepidium draba*, *Sinapis alba*, *Urtica dioica* and *U*. *pilulifera* are only fried. Additionally, the leaves of *Allium ampeloprassum*, stems of *Gundelia tournefortii* and the aerial parts of *Amaranthus retroflexus*, *Anchusa strigose*, *Chenopodium album*, *Convolvulus arvensis*, *Malva neglecta* and *Polygonaum cognatum* are boiled, added to yogurt and bulgur or boiled and sometimes fried with egg.

Although the consumption of *Anchusa strigosa* is common in the study area, the use of this plant as food is not common in Turkey [26, 31, 94] and the other regions that it distributed. The young leaves of *Anchusa strigosa* are first boiled and then fried with onion. The species is also used for medicinal purposes [112–116]. It contains alkaloids, tannins and vitamin E [117]. Young stems, roots and petioles of *Gundelia tournefortii* are consumed raw or cooked in the study area, as in the other parts of Turkey [26, 27, 31, 33, 40–42, 52, 55, 60, 65, 70, 72, 78, 80–82, 84, 86, 89, 94] and in bordering countries [96, 98, 99, 114, 118]. This species is a good source of vitamins A, C and E [119].

Two recent studies [44, 45] documented the taxa used for preparing ‘Sarma’ in Turkey and the Balkans. Sarma is a cooked leaf rolled around a filling made from rice and/or minced meat. *Rumex obtusifolia* subsp. *subalpinus* and *R*. *crispus* are among the plants used for preparing sarma in the study area.

It is common to produce herbal cheese among the local people in the eastern and the southeast regions of Turkey, where ovine are bred and aromatic herbaceous plants are widely grown [74]. The leaves of *Allium ampeloprassum*, *A*. *chloranthum* and *A*. *wendelboanum* are eaten fresh or added to cheeses. To produce this herbal cheese, once the sheep milk is fermented and gelatinised, a coating of gel is placed in a cloth, a layer of milk is placed in the gel, a layer of finely divided chopped plant pieces is added, the bag is tightly wound and a weight is placed on the bag to drain water. *Allium ampeloprassum* is often used for the same purpose [60, 70, 74], but the use of *Allium chloranthum* and *A*. *wendelboanum* is not mentioned previously in the ethnobotanical studies in Turkey.

In the Mediterranean region and similarly in the study area, the immature flower buds of *Capparis* species are often used to prepare pickle [2, 3]. Also it is used as pickle and food in Cizre and Şanlıurfa and in some other regions of Turkey [27, 28, 41, 52, 56, 65].

Even though *Hirschfeldia incana* is broadly distributed in Turkey and was reported in an ethnobotanical study in Iran [120], its consumption was not previously reported. This species is consumed as *Sinapis* and *Lepidium* species.

**Fig. 7:**
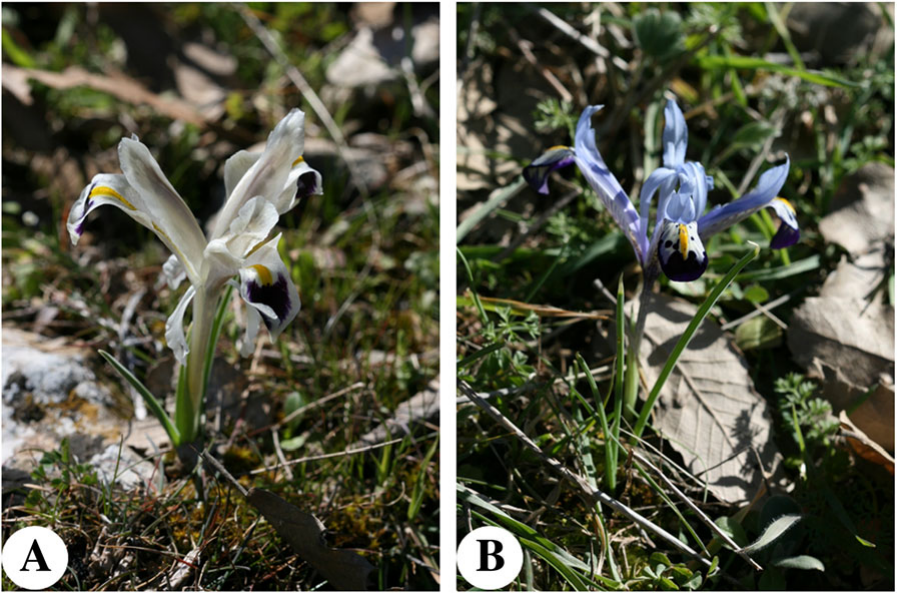
Flowers of *Iris* species consumed by people in the present study. **a** *Iris persica*. **b** *I*. *reticulata*

*Iris persica* and *I*. *reticulate* are known as the heralds of the arrival of spring. Their flowers are eaten raw and have a mild taste (Fig. 7). The same consumption was reported previously for *Iris persica* [82, 94] and for *Iris reticulate* [82]. However, it was stated that the infusion prepared from flowers of *Iris reticulate* was consumed like tea in Midyat [31]. Also, the petals of *Rosa foetida* are eaten raw by children. The consumption of the flowers of this plant as food and for medicinal purposes has been recorded in Midyat [31], but there are no previous records of the use of flowers as food.

**Fig. 8:**
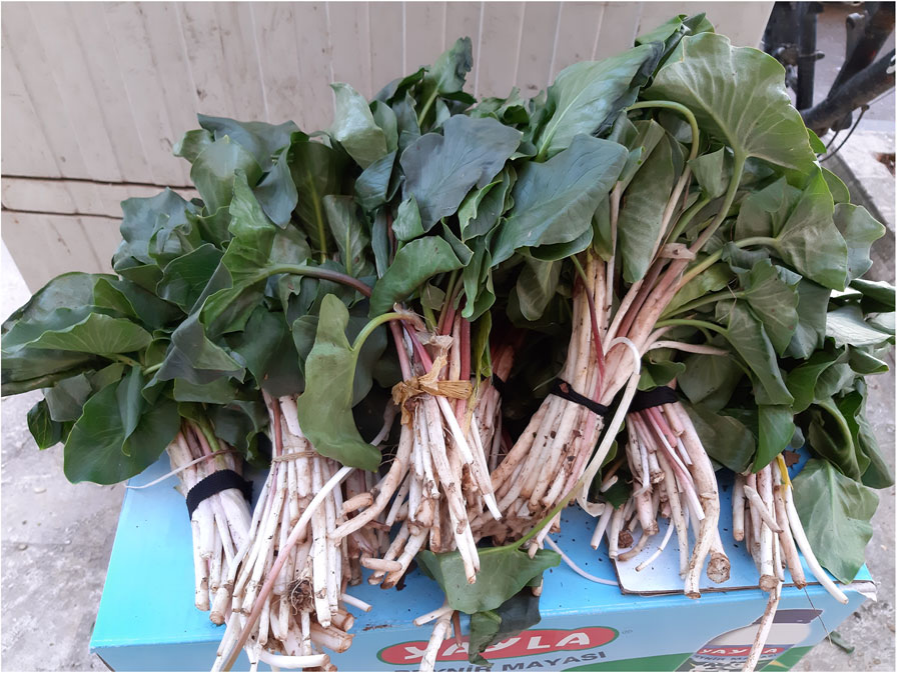
*Arum rupicola* leaves in the local market in Yeşilli District

Previous studies reported that *Arum* spp. leaves were kept in an acidic environment (created using a powdered sour sumac spice mixed with water or cheese water) before the leaves were consumed to remove excess oxalates and sharp flavour found in the leaves [94, 96, 98, 99]. In the present study, lemon salt and pomegranate juice were also mentioned in addition to sumac spice by the interviewees for detoxification of *Arum rupicola* leaves (Fig. 8). After this process, the boiled leaves are fried with onion.

*Euphorbia* species are often known as poisonous and they are not consumed directly. However, the aerial parts of *Euphorbia helioscopia* subsp. *helioscopia* are added to molasses white soil when it boils to improve the test of molasses. Also the consumption of this species was reported previously as food [41]. Moreover, it was reported that *Euphorbia* species were used to clean thick buble layer on grape molasses in Midyat [31], and were added to the jam and used to ferment cheese or dough in Cizre [52].

### Fruits

The fruits of 13 taxa are consumed raw. However, the fruits of *Celtis tournefortii*, *Ficus carica* subsp. *carica* and *F*. *carica* subsp. *rupestris* are also dried and eaten in winter. The most common fruits consumed by informants were *Celtis tournefortii*, *Cerasus prostrata*, *Crataegus azarolus* var. *azarolus*, *Elaeagnus angustifolia*, *Ficus carica* subsp. *carica*, *Rosa canina* and *Rubus sanctus*. *Ficus carica* subsp. *carica* had the highest CI index in the study area; the consumption of this taxa is commonly reported [20, 26, 37, 38, 40, 43, 50, 52, 55, 56, 58, 59, 62, 63, 72, 75, 82, 86, 92, 96]. The consumption of the fruits of *Ficus carica* subsp. *rupestris* is not common [27, 40, 94]. *Rosa canina* has high local value as a fruit in Turkey, as well as in bordering countries and European countries [2–4, 20, 32–36, 38, 40, 41, 47–49, 51–53, 55, 60, 62, 63, 65, 69–72, 74, 75, 78, 81, 82, 86, 93, 94, 96]. The fruits of this species are well-known for their efficacy in strengthening the body’s defence against infection and particularly the common cold [36]. Furthermore, fruits are rich in sugar, pectin, organic acids, essential oils, tannins and minerals (P, K, Mg, Ca, Fe, Mn) [121]. The fruit of *Cerasus microcarpa* subsp. *microcarpa* are eaten, and the same usage was observed in previous studies [49, 60, 94]. However, the consumption of *Cerasus prostrata* var. *prostrata* was not previously reported. The fruits of *Celtis tournefortii* are consumed freshly; additionally, the mature fruits have another interesting usage in the region. The fruits are fried, crushed and powdered and used to prepare a mixture with honey or molasses, which is usually eaten in winter. *Celtis tournefortii* fruits have important antiradical, antimicrobial and antiproliferative properties [122]. The similar use of *Celtis glabrata* Steven ex Planch. fruits has been previously reported [60].

### Seeds

The seeds of five taxa from the Fabaceae family are eaten raw. The local name ‘colban’ is used for the fruits of *Vicia* and *Lathyrus* species in the southeast of Turkey [27]. However, these plants named ‘baqil’ in Yeşilli. Also, they are named according to the shapes of the fruits, seeds and leaves in the study area. *Baqil* is the common name of these species. *Vicia palaestina* is called baqilê çûkan; ‘*çûkan*’ means ‘*birds*’ and this name is used because the fruit of this plant is smaller than the fruit of other *baqil* species. *Vicia pannonica* is called baqilê gan; ‘*gan*’ means ‘*ox's*’, and this name is used because the fruit of this plant is larger than the fruit of other *baqil* species. The usage of these taxa as only fodder was reported in Urfa [26]. *Vicia pannonica* var. *pannonica* is commonly used according to an ethnobotanical study conducted in Midyat district [31].

*Vicia sativa* is named baqilê maran; ‘*maran*’ means ‘*snake's*’ and this name is used because the fruit of the plant is longer and thinner than the fruit of other *baqil* species. The consumption of the fruits of *Vicia sativa* was previously reported [27, 49, 52, 63]. The seeds of this plant contain 31% crude protein, 327% starch, 54% sugar and 35–44% fibre, cellulose and lignin [107].

Moreover, *Lathyrus cassius* is named baqilê bizinan; ‘*bizinan*’ means ‘*goat's*’, as the fruits and leaves of this plant are smaller than the fruit of *Vicia pannonica*. *Pisum fulvum* is named ‘baqilê xatȗnȋ’; ‘*xatȗnȋ*’ means ‘goddess's’, the seeds of this plant are tastier than those of other *baqil* species. The consumption of *Lathyrus cassius* and *Pisum fulvum* were reported for the first time in this study.

The fruits of *Quercus brantii* were reported to be eaten raw and roasted in embers in the present study, but raw consumption is generally reported for medicinal purposes in Turkey [31, 40, 52, 61]. A previous study reported the same consumption in the southeast of Turkey [123].

### Beverage

**Fig. 9:**
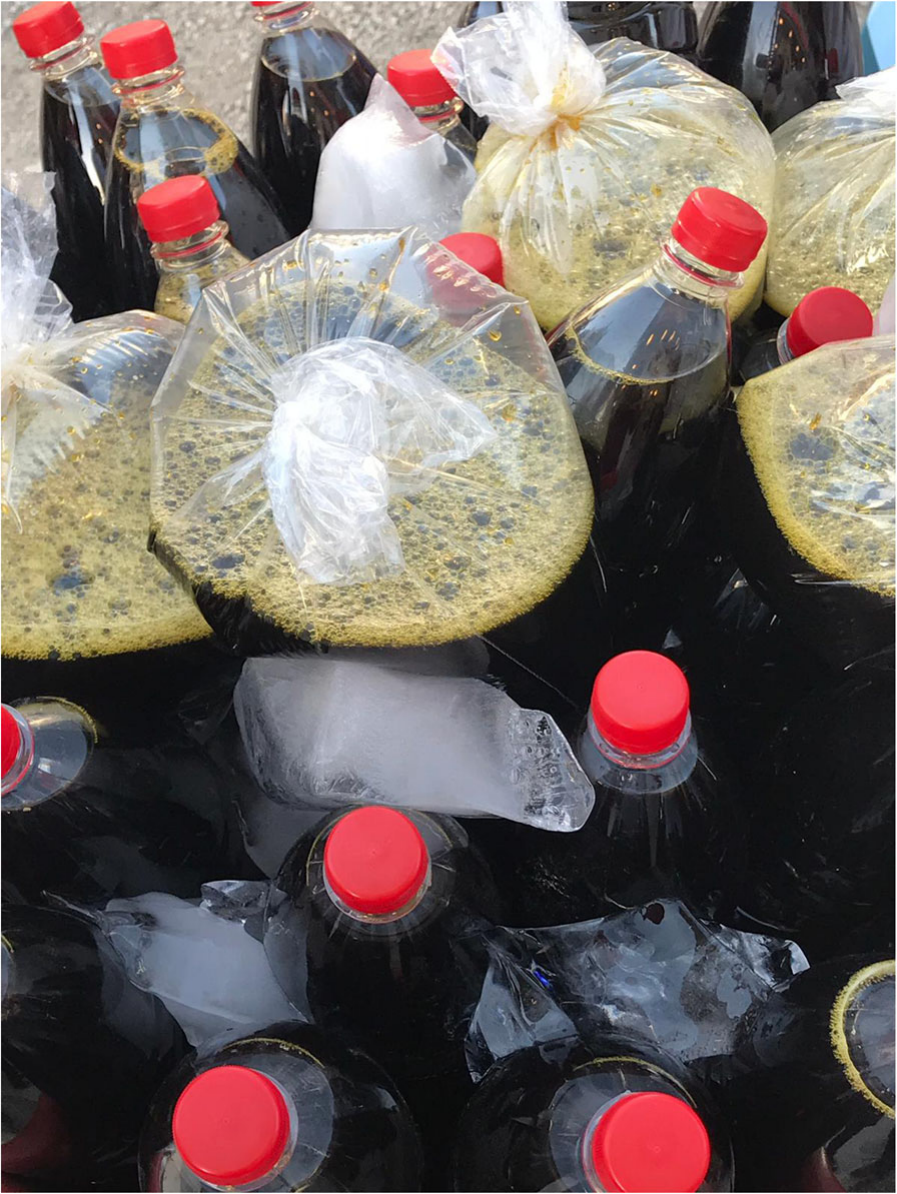
Liquorice syrup obtained from *Glycyrrhiza glabra* roots in a shop in the center of Yeşilli

In the studied area, five taxa were reported to be used for preparing beverages such as herbal teas, coffees, liqueur or wine and cold drinks. The flowers of *Elaeagnus angustifolia* and the leaves of *Mentha longifolia* are prepared as a herbal tea. Immature fruits of *Pistacia terebinthus* subsp. *palaestina* are collected when they are purple and blue (in September). Then, these fruits are dried, crushed and cooked as coffee. This coffee is called *melengiç, menengiç, çitlenbik, çedene, kizvan kahvesi* and is prepared with water or milk. It is very common in Turkey’s southeast region and is consumed daily as a drink [30, 38, 41–43, 49, 50, 52, 54, 56, 59, 60, 65, 75, 78, 81, 86, 91]. The Christian people use the fruits of *Cerasus mahaleb* var. *mahaleb* to prepare liqueur and wine. The roots of *Glycyrrhiza glabra* are used to prepare a traditional cold beverage called ‘ava susê’ during summer. To prepare *ava susê*, the collected roots are washed, cut to a length of 20 cm, placed in a bowl and sprinkled with a small amount of water. Then, the mixture is kneaded like dough. When the water is absorbed, more water is added. This process is repeated several times. Additional water is added to the roots. The liquorice root water obtained is called yeast. A little more water is added to the yeast, and liquorice syrup is obtained (Fig. 9). To remove the bitter taste, the syrup is poured from one container to another and the foam is cleared. *Ava susê* has an important place in folk culture and there is even a Kurdish song about this drink in the region. The drink is distributed by itinerant vendors carrying large decorated pitchers on their backs. The consumption of *Glycyrrhiza glabra* roots in this way was also reported in a previous study [12] and it is generally consumed in the eastern and the south-eastern parts of Anatolia [26, 27, 32, 41, 48, 52, 78, 80–82, 93]. The sweet taste of the beverage is due to the substances in it. *Glycyrrhiza glabra* contains flavonoids, isoflavones, saponins, coumarins, chalcones, sterols and alkaloids [124]; the main component glycyrrhizin (2–25% content) is 50 times sweeter than sucrose [124]. The plant is used to treat colds [52, 77], respiratory tract diseases, flu, bronchitis, gastrointestinal diseases, smoking addiction [83] and diabetes [90], and as analgesic. Antioxidant, antimicrobial (especially against *Helicobacter pylori*), immunostimulant and anticancer effects have also been reported in this plant [124].

### Spices

Because of their aromatic properties, species from the Lamiaceae family, including *Mentha longifolia*, *Thymbra spicata* and *Thymus kotschyanus* subsp. *kotschyanus*, are used as spices; the aerial parts of these species are collected in April and are dried in a shady place and used as spices. *Mentha longifolia* is a valuable spice in Turkey [32–34, 38, 40, 44, 47–49, 51–54, 58, 60, 62, 63, 65, 69, 70, 72, 74–76, 78, 80–82, 84–89, 94] and close regions of bordering countries [94, 96, 98, 99].

The mature fruits of *Rhus coriaria* are collected at the end of July or in the first days of August and dried in shady places. The dried fruits are then crushed. *Rhus coriaria* is usually used where it is distributed [20, 28, 31, 34, 38, 41, 52, 55, 60, 65, 66, 71, 75, 78, 81, 82, 86, 92, 96]. The common name of *Rhus coriaria* is sumac; this name derives from ‘sumaga’, which means red in Syriac [125]. It is used by indigenous peoples for medicinal purposes and other uses [126]. Different parts of sumac possess significant phytochemical components, such as tannins, flavonoids, anthocyanins, organic acids, flavones, proteins, fibre, volatile oils, nitrates and nitrites [127]. Furthermore, antimicrobial, antifungal and antiviral activities of *Rhus coriaria* were reported [127]. *Thymbra spicata* and *Thymus kotschyanus* subsp. *kotschyanus* are generally called *cahter* or *cehter*, and they are the leaves are collected before flowering time, dried in the shade place and added to the food as flavour and fragrance. In the literature study, it was observed that *Thymus kotschyanus* subsp. *kotschyanus* was consumed as spices especially in Eastern Anatolia [32, 60, 62, 70, 74, 78, 81, 82]. However, the use of *Thymbra spicata* was reported usally as spice in the Mediterranean and nearby regions [28, 30, 31, 38, 43, 55, 74, 78, 86].

### Nectar and latex

**Fig. 10:**
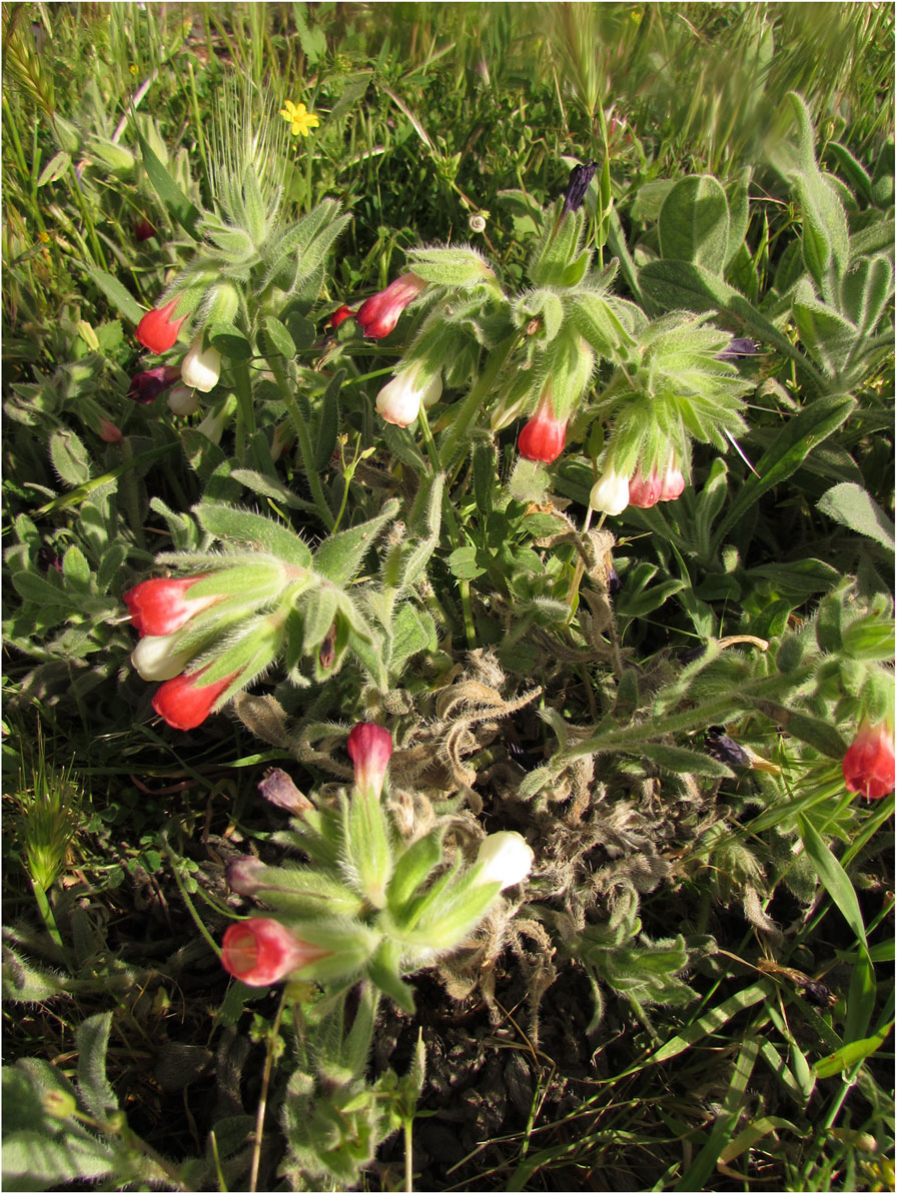
*Onosma alborosea*

**Fig. 11:**
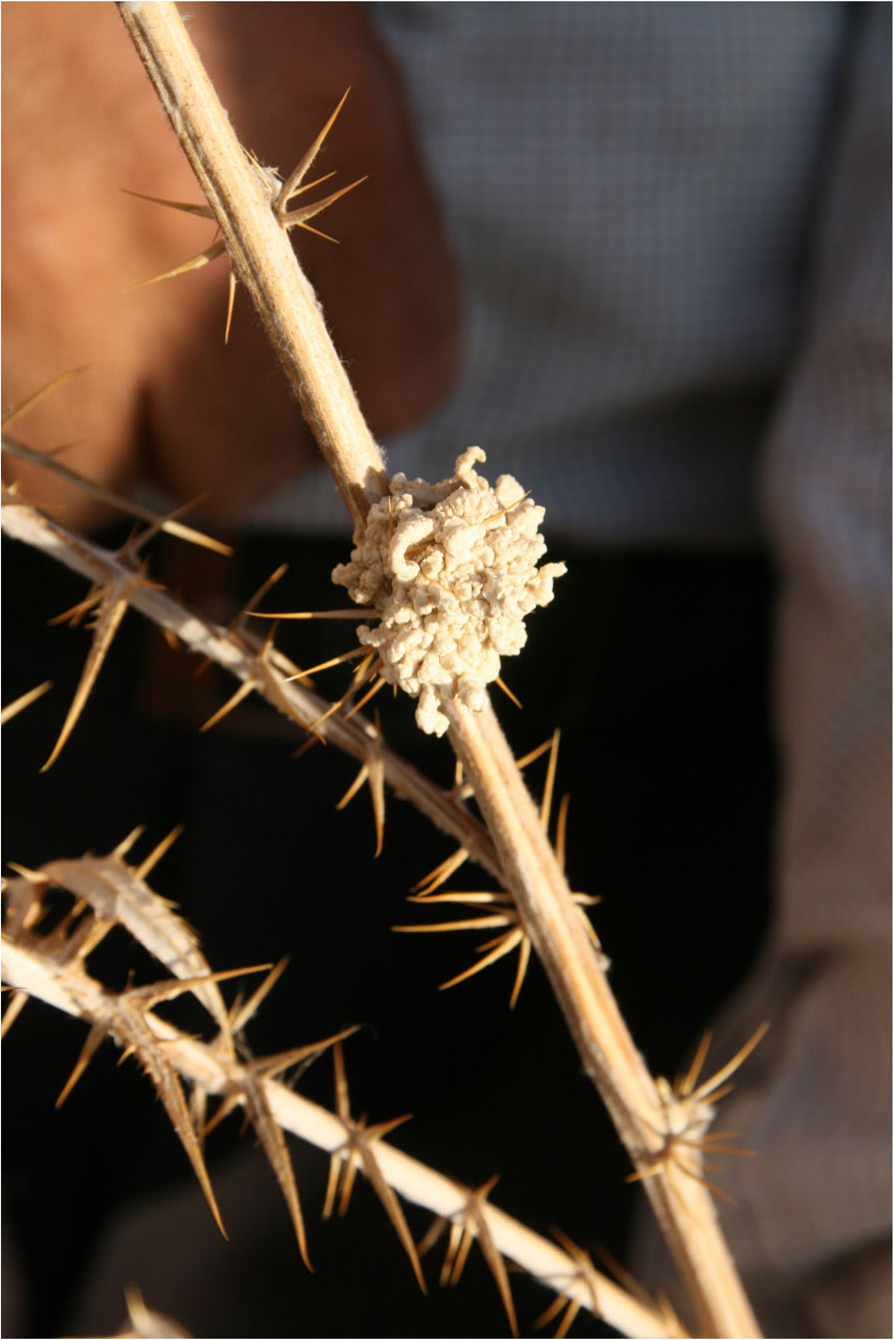
The latex of *Echinops orientalis*

Boraginaceae is known to produce high-quality nectar, which is a sugar-rich solution [128]. Usually, the nectar of flowers of *Anchusa* and *Echium* species is sucked [26, 31, 94, 99]. However, we observed the sucking of the nectar of *Onosma roussaei* and *O*. *alborosea* for the first time (Fig. 10). The edible latex which flows through the stems of *Echinops spinosissimus* subsp. *bithynicus* and *E*. *orientalis* that flows through the stem of these taxa is known as ‘helva’ because of its sweet taste (‘*helva*’ means ‘*sweet*’ in Arabic) (Fig. 11). This consumption of receptacles is common [27, 40, 60, 70, 78, 94], but the consumption of dried latex has not been previously reported.

### Medicinal WEPs

Approximately quarter of the gathered and consumed wild vegetables that were recorded in this study (most notably *Anchusa strigosa*, *Cerasus mahaleb* var. *mahaleb*, *Mentha longifolia*, *Glycyrrhiza glabra*, *Malva neglecta*, *Rosa canina* and *Urtica dioica*) are also used for medicinal purposes; these plants are called nutraceuticals folk functional foods [12]. In some interviews, it was recorded that every eaten plant is believed to be good for health. In the present study, 16 wild edible taxa were reported as food medicine, including wild vegetables, roots and fruits. The aerial parts of *Urtica dioica* (CI: 0.72) are cooked as vegetables and consumed for the treatment of cancer, rheumatism and diabetes. *Urtica dioica* is a multi-purpose medicinal species [129]; it is consumed in addition to the fruits of *Rosa canina* (CI: 0.53) for the treatment of colds, flu, cough, urinary tract burning and bronchitis. *Urtica dioica* is a rich source of flavonoids, carotenoids and fatty acids and has a high content of vitamins (especially vitamin C), minerals (Ca, Mg, Fe, Al, Mn, Zn, Sr, Ba, etc.) and antiinflammatory agents [130]. Additionally, some parts of this medicinal plant and other plants (*Cerasus mahaleb* var. *mahaleb*, *Thymbra spicata* and *Quercus brantii*) are collected by local people, and sold in the local bazaars.

## Conclusion

A comparison of the traditional uses of wild edible plants reported, in the literature, revealed that the current study is the first to record the use of *Allium chloranthum*, *A*. *wendelboanum*, *Centaurea virgata*, *Cerasus prostrata* var. *prostrata*, *Gagea villosa*, *Geocaryum cynapioides*, *Lathyrus cassius*, *Onosma alborosea*, *Papaver glaucum*, *Pisum fulvum*, *Rosularia radiciflora* and *Sedum pallidum* in Turkey and bordering countries. The culturally important plants in the study area (*Ficus carica* subsp. *carica*, *Gundelia tournefortii*, *Glycyrrhiza glabra*, *Juglans regia*, *Lepidium draba*, *Malva neglecta*, *Mentha longifolia*, *Rhus coriaria*, *Rosa canina*, *Rumex crispus*, and *Urtica dioica*) are also commonly used in other parts of Turkey and bordering countries near the study area, which shows that the residents of these regions have some similarities in their traditions.

This study in Yeşilli district, which is culturally diverse, and this area has not been subject to any ethnobotanical work previously. In multicultural and multilingual area, there are differences in plant usage as well as in plant names. Therefore, this study has made an important contribution to the preservation of cultural heritage related to traditional wild edible plants in this region. The villagers continue to agriculture and animal husbandry intensively. However, a large part of the population consists of elderly people, middle-aged people and children. The older generations of villagers have extensive knowledge of useful plants, and this knowledge is transmitted orally from one generation to another. Most of the young population works in large cities. Unfortunately, the few young people living in the villages are not interested in learning traditional knowledge from their ancestors. Hence, there is an urgent need to document their folklore, before traditional knowledge becomes unattainable and eventually extinct. Our findings will contribute to increasing the existing knowledge of wild edible plants in Mardin and its surroundings and will be a source for comparative intercultural ethnobotany.

## Data Availability

All data generated or analysed during the conduct and writing up of the manuscript is incorporated in the research article. Voucher specimens were deposited at the Herbarium of Faculty of Pharmacy, University of Istanbul (ISTE).
